# Bone Changes in Mandibular Condyle of Temporomandibular Dysfunction Patients Recognized on Magnetic Resonance Imaging

**DOI:** 10.3390/jimaging12010005

**Published:** 2025-12-24

**Authors:** Fumi Mizuhashi, Ichiro Ogura, Ryo Mizuhashi, Yuko Watarai, Tatsuhiro Suzuki, Momoka Kawana, Kotono Nagata, Tomonori Niitsuma, Makoto Oohashi

**Affiliations:** 1 Departments of Removable Prosthodontics, The Nippon Dental University School of Life Dentistry at Niigata, Niigata 951-8580, Japan; watarai@ngt.ndu.ac.jp (Y.W.); tatsu@ngt.ndu.ac.jp (T.S.); 2 Functional Occlusal Treatment, The Nippon Dental University Graduate School of Life Dentistry at Niigata, Niigata 951-8580, Japan; kawana.momo@ngt.ndu.ac.jp (M.K.); nagatako35@ngt.ndu.ac.jp (K.N.); niitsuma@mx.ngt.ndu.ac.jp (T.N.); 3 Departments of Oral and Maxillofacial Radiology, The Nippon Dental University School of Life Dentistry at Niigata, Niigata 951-8580, Japan; ogura@ngt.ndu.ac.jp; 4 Comprehensive Dental Care, The Nippon Dental University Niigata Hospital, Niigata 951-8580, Japan; ryo-mz@ngt.ndu.ac.jp; 5 Department of Dental Anesthesia and General Health Management, The Nippon Dental University School of Life Dentistry at Niigata, Niigata 951-8580, Japan; oohashi@ngt.ndu.ac.jp

**Keywords:** bone change, magnetic resonance imaging, temporomandibular disorders

## Abstract

We aimed to investigate the type of bone changes in temporomandibular disorder patients with disc displacement. The subjects were 117 temporomandibular joints that were diagnosed with anterior disc displacement using magnetic resonance imaging (MRI). Temporomandibular joint (TMJ) pain and opening dysfunction were examined. Disc displacement with and without reduction, joint effusion, and bone changes in the mandibular condyle were assessed on MRI. The types of bone changes were classified into erosion, flattening, osteophyte, and atrophy on the MR images. Fisher’s exact test and χ^2^ test were performed for analyses. Bone changes were found on 30.8% of subjects with erosion, flattening, osteophyte, and atrophy types (*p* < 0.001). The occurrence of joint effusion appearance (*p* < 0.001), TMJ pain (*p* = 0.027), and opening dysfunction (*p* = 0.002) differed among the types of bone changes. Gender differences were also found among the types of bone changes (*p* < 0.001). The rate of disc displacement with reduction was significantly smaller than that of disc displacement without reduction on flattening and osteophyte (*p* < 0.001). The results made it clear that the symptoms, gender, and presence or absence of disc reduction differed among the types of bone changes.

## 1. Introduction

Temporomandibular disorders (TMDs) are common diseases in dentistry. The World Health Organization (WHO) indicated that TMDs are the third most common dental condition after dental caries and periodontal disease [1]. TMDs affect 34% of the global population [2], and the prevalence rates differ between each continent. Several epidemiologic studies have reported that 40 to 70% of the general population experiences some signs of TMDs [3,4], and about 4 to 7% of the population indicates symptoms of sufficient severity to need treatment [5]. TMDs are common in women compared to men [6], and the peak age of the prevalence of TMDs is from 45 to 64 [7,8].

TMDs show some clinical problems on the masticatory musculature, the temporomandibular joint (TMJ), and associated structures [9]. The three most common symptoms of TMD are pain, joint noises, and disturbance in jaw opening [10,11,12]. Sometimes, the symptom of headaches appears with TMDs [13,14]. Disease states are classified as myofascial pain, arthralgia, disc displacement with or without reduction, and osteoarthrosis [15,16]. Among these disease states, disc displacement is the most common state [17], and some studies have reported the prevalence of disc displacement as 77 to 89% [18], 48.9% [19], or 41% [17]. Disc displacement is the state in which the TMJ loses the normal disc–condyle relationship in the closed-mouth position, and the displacement of the disc to the anterior, posterior, inner, and outer sides of the condyle occurs [20]. Most disc displacements are to the anterior of the mandible condyle [15,16], which causes internal derangement [12]. Overload of the bilaminar zone of TMJ is caused by the direct contact of the condyle with disc displacement, and the load induces pain in the TMJ [21]. The load can be one of the risk factors for osteoarthrosis [22,23]. Osteoarthrosis involves cartilage destruction, osseous degenerative changes, and subchondral bone remodelling [24,25]. Some studies reported that the internal disorder of TMJ, mainly in the case of disc displacement without reduction (DDWOR), causes osteoarthrosis [23,26].

Magnetic resonance imaging (MRI) has been used for the detection of disc displacement. MRI is the gold standard for detecting disc displacement [27] and is necessary for TMD diagnosis [28,29]. Recently, 3D ultrasound has been shown to have acceptable diagnostic efficacy to detect disc displacement with a non-ionizing imaging method, and it is less expensive, transportable, and more comfortable to the patient [9]. It has potential utility in detecting both osteochondral and soft-tissue changes [30]. MRI is effective at characterizing not only the disc morphology or disc position but also the osseous joint morphology [31,32]. On MRI images, the intra-observer agreement for detecting the position of the disc is 95%, and that for osseous changes is 97% [33]. MRI is usually performed with 1.5 T or 3.0 T scanners with a head coil or TMJ surface coil to detect the disc morphology, disc position, and the osseous joint morphology [34]. MRI allows the visualization of hard tissues without exposing the patient to ionizing radiation [35,36], and this is one of the advantages of MRI. Disc displacement and osseous joint morphology can be confirmed at the proton density with high contrast, and the joint effusion is recognized on the T2-emphasized image of MRI. Joint effusion is observed as a hyperintense area on a T2 image, in which synovial fluid is accumulated at the superior and/or inferior articular cavity. Joint effusion reflects the condition of inflammation in TMJ [37].

Previous reports showed that DDWOR occurs as osteoarthrosis [23,26], and a nine-times-greater likelihood of osteoarthrosis occurrence was observed in DDWOR compared to the disc displacement with reduction (DDWR) [28]. Osteoarthrosis shows erosion, flattening, osteophytes, irregularities, or deformities on the surface of the mandibular condyle, as well as subchondral bone sclerosis [38]. However, few reports show the relation between the type of bone changes and the symptoms [39]. This study aimed to examine the type of bone changes in disc displacement and mentions the factors relating to bone changes.

## 2. Materials and Methods

This study was conducted by reviewing the MRI of TMD patients who came to the Temporomandibular Joint Disorders and Bruxism Clinic, The Nippon Dental University Niigata Hospital (Niigata, Japan). We subjected 117 TMJs of 65 TMD patients (24 male, 93 female, mean age 43.7 ± 18.4 years) who were diagnosed with TMD with anterior disc displacement (with or without reduction) using MRI. Clinical presentation for TMJ pain was interviewed. As a clinical examination, the distance of the mouth opening was measured to check the opening dysfunction. The opening dysfunction was decided when the distance of the mouth opening was smaller than 40 mm, because the opening dysfunction by the DDWOR is defined as smaller than 40 mm by the Japanese Society for Temporomandibular Joints. The distance of mouth opening was decided by measuring the distance between the incisal edge of the upper and lower right central incisors using a calliper and subtracting the overbite distance from the inter-incisal edge distance. After the clinical presentation and the clinical examination, image examination was conducted using MRI. This retrospective study was approved by the ethics committee of our institution.

MRI (1.5 Tesla MR unit; EXCELART VantageMRT-2003; Canon Medical Systems, Otawara, Japan) was used with a surface coil for the TMJ, including proton-density-weighted imaging on the positions of mouth closing and maximum opening of mouth (repetition time/echo time: 2000 ms/18 ms, field of view: 130 mm × 130 mm, matrix size: 256 × 224, 1 acquisition). Additionally, T2-weighted imaging on the mouth closing and maximum opening of the mouth (repetition time/echo time: 3500 ms/100 ms, field of view: 130 mm × 130 mm, matrix size: 256 × 192, 2 acquisition) was included [35,40,41]. MRI images were independently evaluated by two specialists in radiology, and any discrepancies were resolved by consensus. The positions of the disc were confirmed on the proton-density-weighted sagittal imaging, and the TMJs with anterior disc displacement were enrolled in this study. The anterior disc displacement was further classified into DDWR and DDWOR.

Joint effusion was detected on T2-weighted imaging of MRI with hyperintense areas in the articular cavities. Bone changes in the mandibular condyle were also assessed on proton-density-weighted sagittal imaging of MRI. The type of bone changes was classified into erosion, flattening, osteophytes, and atrophy, as stated in the Japanese Society for Temporomandibular Joints (Table 1). When the multiple co-existing bone changes were recognized, the classification was decided based on the one in which the characteristics were clearer by the two specialists of radiology.

Statistical analyses were performed using χ^2^ tests for analyzing the rate of the type of bone changes, the rate of joint effusion, the rate of TMJ pain, and the rate of opening dysfunction at each type of bone change. The proportion of the number of males and females with each type of bone change was analyzed by Fisher’s exact test, and the post hoc test was performed by residual analysis. Additionally, the proportion of the number of subjects on DDWR and DDWOR with each type of bone change was also analyzed by Fisher’s exact test, and the post hoc test was performed by residual analysis. Analyses were conducted using software (SPSS 17.0, SPSS JAPAN, Tokyo, Japan), and differences in α < 0.05 were considered significant.

## 3. Results

**Figure 1:**
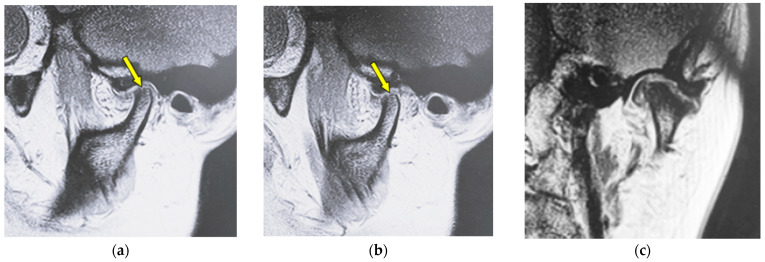
MR image (proton-density-weighted) of a 43-year-old woman (disc displacement without reduction, left disc): (**a**) sagittal oblique cross-section imaging in the mouth-closing position; (**b**) sagittal oblique cross-section imaging in the mouth-opening position; (**c**) coronal oblique cross-section imaging in the mouth-closing position. The arrow shows the part with erosion.

Figure 1 shows MR images of a 43-year-old woman with erosion (left TMJ). Sagittal oblique cross-section imaging (proton-density-weighted) shows anterior disc displacement at the mouth-closing position (Figure 1a) and at mouth-opening positions (Figure 1b).

**Figure 2:**
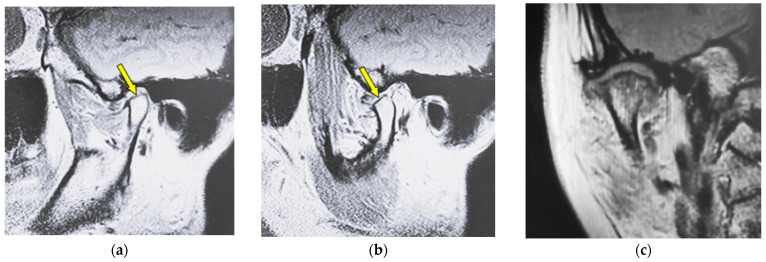
MR image (proton-density-weighted) of a 51-year-old woman (disc displacement without reduction, right disc): (**a**) sagittal oblique cross-section imaging in the mouth-closing position; (**b**) sagittal oblique cross-section imaging in the mouth-opening position; (**c**) coronal oblique cross-section imaging in the mouth-closing position. The arrow shows the part with flattening.

Figure 2 shows MR images of a 51-year-old woman with flattening (right TMJ). Sagittal oblique cross-section imaging (proton-density-weighted) shows anterior disc displacement in the mouth-closing position (Figure 2a) and in the mouth-opening position (Figure 2b).

**Figure 3:**
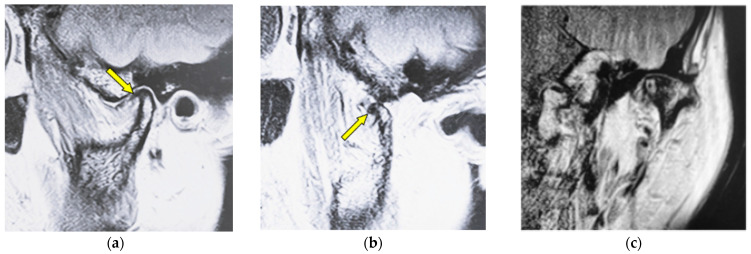
MR image (proton-density-weighted) of a 53-year-old woman (disc displacement without reduction, left disc): (**a**) sagittal oblique cross-section imaging in the mouth-closing position; (**b**) sagittal oblique cross-section imaging in the mouth-opening position; (**c**) coronal oblique cross-section imaging in the mouth-closing position. The arrow shows the part with an osteophyte.

Figure 3 shows MR images of a 53-year-old woman with osteophyte (left TMJ). Sagittal oblique cross-section imaging (proton-density-weighted) shows anterior disc displacement in the mouth-closing position (Figure 3a) and in the mouth-opening position (Figure 3b).

**Figure 4:**
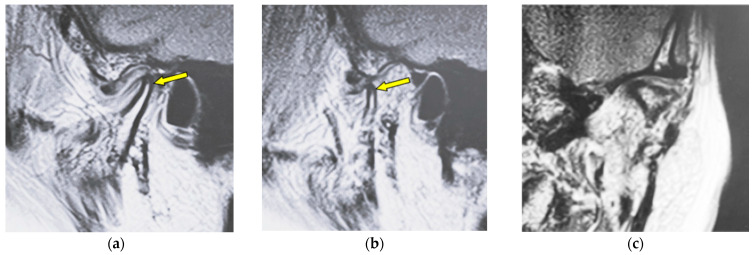
MR image (proton-density-weighted) of an 82-year-old woman (disc displacement without reduction, left disc): (**a**) sagittal oblique cross-section imaging in the mouth-closing position; (**b**) sagittal oblique cross-section imaging in the mouth-opening position; (**c**) coronal oblique cross-section imaging in the mouth-closing position. The arrow shows the part with atrophy.

Figure 4 shows MR images of an 82-year-old woman with atrophy (left TMJ). Sagittal oblique cross-section imaging (proton-density-weighted) shows anterior disc displacement in the mouth-closing position (Figure 4a) and in the mouth-opening position (Figure 4b).

Characteristics of the subjects are shown in Table 2. Of the 117 TMJs, 69.2% showed no changes on the mandibular condyle and 30.8% showed changes in the bone such as erosion, flattening, osteophyte, and atrophy. The rates of TMJs with no changes, erosion, flattening, osteophyte, and atrophy were significantly different (χ^2^ (4) = 151.00, *p* < 0.001). There were nearly twice as many female subjects as males for each type of bone change. The mean ages of the subjects with erosion and flattening were higher than those with osteophytes, atrophy, and no changes.

The rates of joint effusion, TMJ pain, and opening dysfunction for each type of bone change are shown in Table 3. The appearance of joint effusion differed by the type of bone changes (χ^2^ (4) = 34.45, *p* < 0.001). Joint effusion appeared in all cases of atrophy. In contrast, joint effusion was found in only 33.3% cases of erosion. The appearance of TMJ pain differed by the type of bone changes (χ^2^ (4) = 10.95, *p* = 0.027). TMJ pain appeared in 62.5% of osteophyte cases, and it appeared in only 33.3% of erosion cases. The appearance of opening dysfunction differed by the type of bone changes (χ^2^ (4) = 16.93, *p* = 0.002). Opening dysfunction was recognized in 87.5% of osteophyte cases and only 44.4% of atrophy cases.

**Figure 5:**
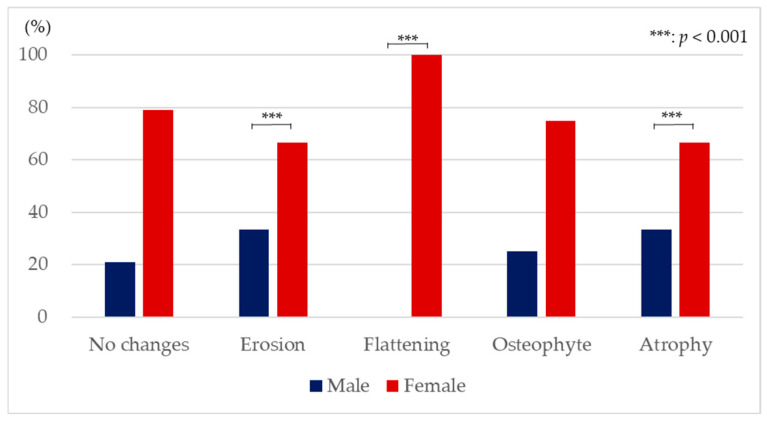
Proportion of the number of males and females with each type of bone change (Fisher’s exact test and residual analysis).

Figure 5 shows the proportion of the number of males and females for each type of bone change. Flattening was observed in only females, and erosion and atrophy were recognized more in females compared to males (χ^2^ (4) = 42.30, *p* < 0.001). Statistically significant gender differences were not found when a bone change did not occur and when there were osteophytes.

**Figure 6:**
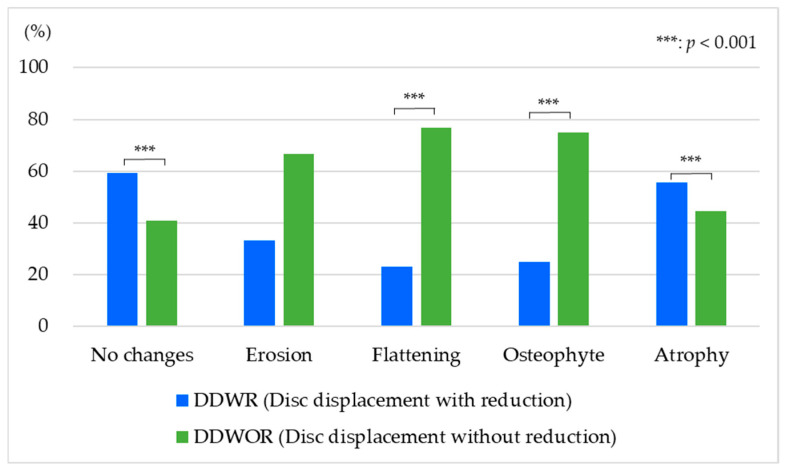
Proportion of the number of subjects with disc displacement with reduction and that with disc displacement without reduction for each type of bone change (Fisher’s exact test and residual analysis).

Figure 6 shows the proportion of the number of subjects with DDWR and those with DDWOR for each type of bone changes (χ^2^ (4) = 49.38, *p* < 0.001). The rate of DDWR was significantly larger than that of DDWOR on the condition that the bone change did not occur (DDWR: 59.3%, DDWOR: 40.7%). The rate of DDWR tended to be smaller than that of DDWOR on erosion (DDWR: 33.3%, DDWOR: 66.7%). The rate of DDWR was significantly lower than that of DDWOR on flattening (DDWR: 23.1%, DDWOR: 76.9%). The rate of DDWR was also significantly lower than that of DDWOR on osteophyte (DDWR: 25.0%, DDWOR: 75.0%). The rate of DDWR was significantly larger than that of DDWOR with atrophy (DDWR: 55.6%, DDWOR: 44.4%).

## 4. Discussion

This study investigated the type of bone changes in TMD patients with disc displacement and examined the relation to the symptoms, gender, and presence or absence of reduction. Subjects over a wide range of ages were included in this study because a significant association between DDWOR and bone changes has been reported in children and adolescent patients with TMD [42]. DDWR is the condition in which the articular disc loses the normal disc–condyle relationship in the closed-mouth position and the disc is in the normal disc–condyle relationship in the opened-mouth position. On the other hand, DDWOR is the condition in which the articular disc loses the normal relationship between the disc and the condyle in both the mouth-closing position and the mouth-opening position. The articular disc plays an important role as a cushion for the condyle and fossa. Under the condition of disc displacement, the cushion cannot work to reduce the impact between the condyle and the fossa, so the internal derangement can easily occur [12]. Therefore, disc displacement leads to symptoms such as TMJ pain, noises, and disturbance of jaw opening and can progress to condylar degeneration [43]. These symptoms appear to worsen with function [44], and the disease state TMD causes the deterioration of quality of life (QOL).

The load imposed on the TMJ can be handled when the articular disc is in its normal physiological position between the mandibular bone surface and the temporal bone surface [45]. However, in the cases that the articular disc is displaced, the load imposed on the TMJ cannot be controlled, which would then lead to osteoarthrosis [37,46]. In this study, TMD patients with disc displacement were examined; 69.2% showed no changes on the mandibular condyle, and 30.8% showed bone changes (Table 2). It is expected that 30.8% of the TMD patients with bone changes will progress from the condition of disc displacement, and the other 69.2% of the TMD patients without bone changes could show bone changes in the years ahead. Some treatment would be needed for TMD with disc displacement to prevent or delay the progress to osteoarthrosis.

Joint effusion has been reported to indicate intra-articular inflammation or synovitis with internal derangement [47] and osteoarthrosis [43]. In this study, joint effusion was observed in 64.2% of TMJs without any bone changes. Joint effusion appearance differed by the type of bone changes, and joint effusion appeared in all cases of atrophy, 69.2% of flattening, 62.5% of osteophyte, and 33.3% of erosion (Table 3). Besides erosion, joint effusion appeared in more than half of the cases of TMJ with disc displacement. This result suggests that the appearance of joint effusion differs by the type of bone changes, and it can easily appear in cases of disc displacement.

The appearance of TMJ pain differed by the type of bone changes, and TMJ pain appeared in 62.5% of osteophytes, 46.2% of flattening, 44.4% of atrophy, and 33.3% of erosion. TMJ pain was also found in 39.5% of TMJ without any bone changes (Table 3). The load to the TMJ would be larger in the case of disc displacement, and then, the TMJ pain would easily appear in cases of disc displacement. It was suggested that TMJ pain can easily appear in the case of disc displacement, and the tendency would be stronger in the case of disc displacement with bone changes. There are several reports that showed a positive relationship between joint effusion and TMJ pain [48]. In this study, the TMJ pain was not recognized in as many TMJs as the appearance of joint effusion. In a future study, the relation between joint effusion and TMJ pain with an increased number of subjects should be investigated to analyze the causes of joint effusion and TMJ pain in osteoarthrosis.

The appearance of opening dysfunction differed by the type of bone changes and was found in 87.5% of osteophyte cases, 76.9% of flattening cases, 66.7% of erosion cases, and 44.4% of atrophy cases. Opening dysfunction was also found in 59.3% of TMJ cases without any bone changes (Table 3). This tendency was similar to that of TMJ pain, and TMJ pain and opening dysfunction easily appeared on osteophytes. In the limit of this study, it was suggested that osteophyte would be related to the appearance of symptoms such as TMJ pain and dysfunction in moving. Further investigation would be necessary to make the relationship between the symptom and bone changes clear.

Gender differences were found both in the condition where bone changes did not occur and in the condition with bone changes. The rate in females was more than twice that in males in all conditions. This result agreed the previous reports [6]. The reason for gender differences in the prevalence of TMD would be related to social factors, differences in pain sensitivity, and health-seeking behaviours [49,50]. Additionally, females are more susceptible because the influence of hormones and physiologic differences between genders would be related [51]. The result of this study indicated that the bone changes in TMJ also easily occurred in females.

The rate of DDWR was significantly lower than that of DDWOR on flattening and osteophyte. Four cases showed multiple co-existing bone changes (flattening and osteophytes, flattening and erosion, osteophytes and erosion, and osteophytes and atrophy), and the classification was shown to be the one in which the characteristics were clearer. The rate of DDWOR was significantly lower than that of DDWR with atrophy and the condition without any bone changes. The unexpected finding that atrophy occurred more frequently with DDWR than DDWOR deserves further investigation. This may reflect different biomechanical loading patterns or distinct pathophysiological pathways. Future studies with larger sample sizes and longitudinal designs are needed to clarify these relationships. It was suggested that changes in the mandibular condyle shape would easily occur with the progress from DDWOR. This result supports the previous reports that mentioned that DDWOR causes osteoarthrosis [23,26,27]. Additionally, the results of this study suggest the possibility that some types of osteoarthrosis, such as atrophy, could easily appear with DDWR.

A major limitation of this study was that MRI was used for the investigation of the type of bone changes in TMD patients. The main strength of MRI is the detailed illustration of soft tissue structures, and it also enables the visualization of bone [52,53]. Computed tomography (CT) and cone-beam computed tomography (CBCT) enable a more precise analysis of the osseous components [54,55]. Concerning MRI, T1-weighted 3.0-T MRI can quantify the cortical bone cross-sectional area with a high resolution in comparison with quantitative CT [56], and the delineation of the cortical bone on TMJ is significantly better in images derived by a 3.0-T MRI rather than a 1.5-T MRI [57]. The MRI used in this study was a 1.5-T MRI, which is not enough to observe bone structure in detail, and the diagnostic accuracy might be lower compared to that using CT, CBCT, and 3.0-T MRI or 3D ultrasound. MRI images were independently evaluated by two specialists of radiology and lacked the inter- or intra-rater reliability. An additional limitation was the study design. This study was retrospective and investigated only anterior disc displacement joints at the single-centre clinic. This study was evaluated with small subgroup sizes and the inclusion of two joints from some patients; therefore, there is a possibility that the reproducibility would be low, and it appears difficult to perform sufficiently robust comparisons between individual types of condylar bone changes to draw meaningful conclusions or clinical relevance in this study. The asymptomatic population was not included in this study, so it cannot show a cause-and-effect relationship. TMJ pain was determined only by interviews in this study; therefore, there is a possibility that the assessment of the TMJ pain differed from that using standardized criteria. Thus, this study has limitations, including the retrospective design, the use of 1.5 T MRI rather than 3 T or CT, and relatively small subgroup sizes. These findings should be considered preliminary and warrant validation in larger prospective studies.

This study investigated the type of bone changes in TMD patients with disc displacement, and the relations to symptoms, gender, and the presence or absence of reduction were analyzed. The results suggested that symptoms, gender, and the presence or absence of reduction differed by the type of bone changes. It was considered that bone change appearances varied among the TMJs, and the diagnosis of osteoarthrosis using MRI is very important to grasp bone changes. In future research, the bone changes on mandibular condyles should be investigated with more subjects, and the mechanism of the occurrence of the difference between bone change types should be clarified.

## 5. Conclusions

This study investigated the type of bone changes in TMD patients with disc displacement using MRI and found that 69.2% showed no changes in the mandibular condyle and 30.8% showed bone changes. Erosion, flattening, and atrophy were recognized more in females compared to males. Flattening and osteophyte easily occur on disc displacement without reduction. Joint effusion appeared in all cases of atrophy, and the symptoms of TMJ pain and opening dysfunction easily appeared in osteophytes.

## Figures and Tables

**Table 1:** Type of bone changes in the mandibular condyle.

Type of Bone Changes	Conditions
Erosion	Local area of rarefaction in compact bone with a lack of cortical bone continuously
Flattening	Loss of convexity of the condylar head outline and keeping the cortical bone continuously
Osteophyte	Local forward outgrowth of the condyle bone with an acute angle from the top of the head of the mandible
Atrophy	Reduction in anteroposterior widths in the mandibular condyle without a round form

**Table 2:** Characteristics of the subjects in this study.

Characteristics	No Changes	Erosion	Flattening	Osteophyte	Atrophy
Number	81	6	13	8	9
Gender					
Male	17	2	0	2	3
Female	64	4	13	6	6
Age					
Mean ± S.D.	41.8 ± 18.4	59.3 ± 9.7	51.7 ± 16.5	41.0 ± 19.1	41.9 ± 16.7
Min	13	47	29	15	18
Max	79	78	79	64	71

S.D.: Standard deviation.

**Table 3:** The rate of joint effusion, TMJ pain, and opening dysfunction at each type of bone change.

Type of Bone Changes	No Changes	Erosion	Flattening	Osteophyte	Atrophy	*p* Value
Joint effusion (%)	64.2	33.3	69.2	62.5	100.0	<0.001
TMJ pain (%)	39.5	33.3	46.2	62.5	44.4	0.027
Opening dysfunction (%)	59.3	66.7	76.9	87.5	44.4	0.002

TMJ: Temporomandibular joint.

## Data Availability

The original contributions presented in this study are included in the article. Further inquiries can be directed to the corresponding author.
